# Association between Guillain-Barré syndrome and SARS-CoV-2 virus infection, including the impact of COVID-19 vaccination in the context of the development and general clinical characteristics of the disease

**DOI:** 10.1007/s13365-025-01267-6

**Published:** 2025-07-07

**Authors:** Jakub Sadowski, Joanna Huk, Sylwia Otulak, Jesica Zawiło, Tomasz Klaudel, Mateusz Roszak, Dominik Tenczyński, Rafał Jakub Bułdak

**Affiliations:** 1 Student Scientific Society of Clinical Biochemistry and Regenerative Medicine, Department of Clinical Biochemistry and Laboratory Diagnostics, Institute of Medical Sciences, University of Opole, Oleska 48, 45‑052 Opole, Poland; 2 Department of Clinical Biochemistry and Laboratory Diagnostics, Institute of Medical Sciences, University of Opole, Oleska 48, Opole, 45-052 Poland

**Keywords:** COVID-19, SARS-CoV-2, Guillain-Barré syndrome, Cytokine storm, Molecular mimicry, COVID-19 vaccinations

## Abstract

During the COVID-19 pandemic, a statistically significant increase in the incidence of Guillain-Barré syndrome (GBS) has begun to be observed. This article discusses the impact of immunological processes on structural and functional changes in the peripheral nervous system on the pathogenesis of GBS. The aim of the systematic review is to analyze and discuss available information from the scientific literature regarding a possible clinical relationship between SARS-CoV-2 infection along with vaccination mainly, adenovector and mRNA vaccines and the development of different types of Guillain-Barré syndrome. The review specifically discusses the role of proinflammatory cytokines and “cytokine storm” in patients with COVID-19 and their potential impact on the phenomenon of “molecular mimicry” and the generation of autoantibodies in GBS. This issue has been expanded to include information from studies on the impact of vaccination against SARS-CoV-2 virus and the higher number of observed cases of Guillain-Barré syndrome. Focusing on the characteristics of the methods, materials, results and conclusions, the review finally included 114 publications, like studies, meta-analyses, clinical cases and reviews. The systematic review was conducted using PubMed, Google Scholar, and Elsevier databases. It pointed out the molecular and clinical association between SARS-CoV-2 virus infections and COVID-19 vaccination, in the development of Guillain-Barré syndrome in the context of its clinical course.

## Background

**Fig. 1:**
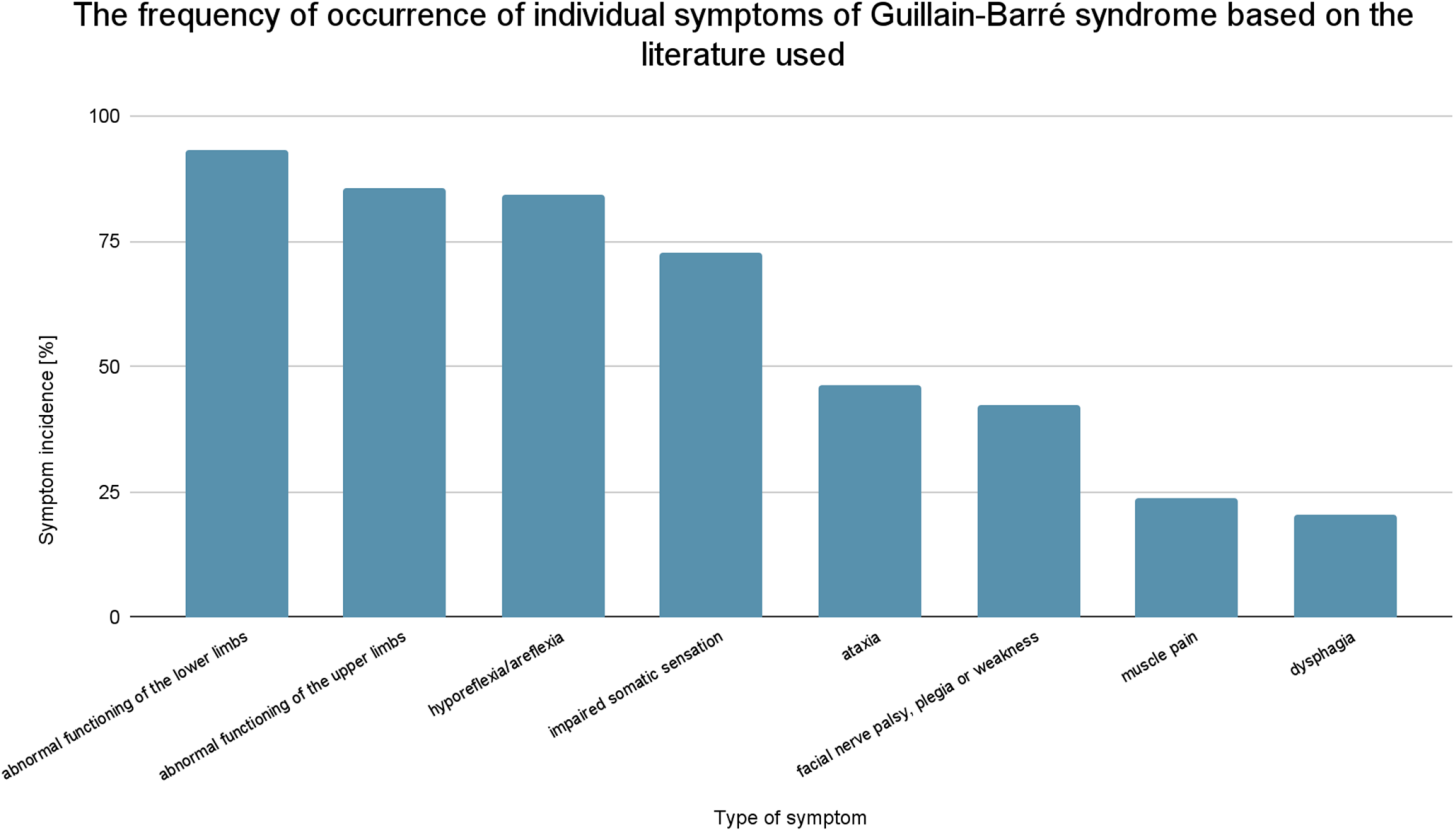
Frequency of occurrence of selected symptoms of Guillain-Barré syndrome according to Bentley et al. (Bentley et al. 2022)

Guillain-Barré syndrome is one of the most common causes of flaccid paralysis worldwide (Shahrizaila et al. 2021). GBS most often manifests itself through progressive weakness of limb, axial, respiratory, or facial muscles with or without concomitant autonomic or sensory impairment (Finsterer 2022; Parry et al. 2007; Versace et al. 2020). Thus, the most common symptoms (based on 147 cases) include: abnormal functioning of the lower limbs (93.2%) and upper limbs (85.7%). In all cases involving both lower and upper limbs, weakness, paresthesia or paralysis occurred (Bentley et al. 2022). The most common neurological symptoms include: hyporeflexia/areflexia (84.4%), impaired somatic sensation (72.8%), ataxia (46.3%), facial nerve palsy, plegia or weakness (42.2%), as well as muscle pain (23.8%) and dysphagia (20.4%) (Fig. 1) (Bentley et al. 2022). This results from the destruction of the myelin sheath by autoantibodies against gangliosides, potentially leading to inflammatory neuropathies (Dardiotis et al. 2020). These autoantibodies are synthesized as a result of the similarity of bacterial and viral antigens to myelin proteins (Shahrizaila et al. 2021; Grygorczuk et al. 2005). This group includes GM1 antibodies (elevated mainly in AMAN– 50–60%) and GQ1b antibodies (elevated in 100% of Bickerstaff brainstem inflammation BBE and Miller-Fisher syndromeMFS cases). In contrast to the above, according to studies, antibodies against GM2, GM3, GM4, GQ2b, antisulfatide, or antititin are statistically usually not elevated (Finsterer 2022). Over the years, the influence of vaccinations used, depending on the pathogen and the technology employed on the development of adverse effects, including GBS, has also been analyzed. The results showed high variability between the studies conducted, with some studies yielding different outcomes. Chen et al. (Chen et al. 2020) reported no significant association between vaccination (hepatitis A/B, influenza, MMR, DTaP, varicella, rabies, live polio, tetanus, Japanese encephalitis, and meningitis) and outcomes in both adult and pediatric cohorts (Chen et al. 2020). However, some studies report a higher number of observed GBS cases following vaccinations, including those against influenza (Goud et al. 2021) and potentially human papillomavirus (HPV) (Martín-Merino et al. 2021). It is noted that in view of the above, the occurrence of Guillain-Barré syndrome in the case of some vaccinations is one of the possible adverse reactions (Sanz Fadrique et al. 2019).

Since 2020, an increase in the incidence of Guillain-Barré syndrome (GBS) has been observed compared to 2019, the period of the pandemic. At the same time, there are more and more reports of a statistical association between the occurrence of GBS in patients after SARS-CoV-2 infection. The COVID-19 pandemic and mass vaccination prompted analyses of potential associations between the rise in GBS cases and vaccination, referencing prior studies on autoimmunity (Sah et al. 2024a, b). An increased risk of the syndrome is suggested in the case of vaccinations using both adenovirus and mRNA, with the risk being higher for the former (Censi et al. 2024). Currently, there is an increase in reports and subsequent studies on this issue. This review summarizes the latest scientific information on the association between COVID-19 and the occurrence of Guillain-Barré syndrome, including its development and clinical characteristics of the disease entity.

### Aim of the study

The aim of the study is to develop, analyze, present and discuss available information within the scientific literature obtained through a systematic review regarding the pathophysiology of Guillain-Barré syndrome in connection with SARS-CoV-2 virus infection. For this purpose, the genesis of the disease was addressed, taking into account clinical subtypes and the role of proinflammatory cytokines in patients with COVID-19. Their influence on the generation of autoantibodies in GBS through the phenomenon of molecular mimicry after a cytokine storm was discussed. Another aim of the study was to discuss the above issue in the context of the impact of immunization during vaccination against SARS-CoV-2 virus based on reports, observations and studies in this area.

## Materials and methods

A systematic literature review was conducted using the following databases: PubMed, Google Scholar and Elsevier without any restrictions. The following keywords and their different combinations were used to search for the materials used: “COVID-19”, “SARS-CoV-2”, “Guillain-Barré syndrome”, “cytokine storm”, “molecular mimicry”; “COVID-19 vaccinations”. During the process, English-language articles were analyzed, including single thematic articles in Polish. The review included publications from 2014 to 2024, including 15 papers from the period before 2014.

The inclusion criteria for the systematic review were as follows: (1) book, original article or review, (2) online document, (3) clinical case report (4) letters to the editor, observational studies, randomized controlled trials and meta-analyses.

The exclusion criteria for the systematic review were as follows: (1) anecdotal, non-updated and unscientific articles (2) articles from before 2014, with the exclusion of selected primary articles in the review topic as part of the search strategy and data collection process (3) studies that did not focus on Guillain-Barré syndrome and COVID-19 infection.

### Search strategy and data collection process

Each author independently conducted a literature review. Initially, a total of 1350 articles from 2014 to 2024 and 320 from earlier years were searched. The above process used a set of keywords: “COVID-19”, “SARS-CoV-2”, “Guillain-Barré syndrome”, “cytokine storm”, “molecular mimicry”; “COVID-19 vaccinations” in various combinations. The search included PubMed, Google Scholar and Elsevier databases. The exclusion criteria were applied and articles meeting them were removed from the database. As a result, 552 articles were excluded. The following filters were applied: meta-analysis, clinical trial, randomized controlled trial, systematic review and online document. 539 items were excluded. In the next step, 185 duplicates were excluded from the collected items. Finally, 394 abstracts were analyzed. 141 articles assessed for eligibility were selected for full content analysis that were relevant to the analyzed topic. The analysis process focused on the characteristics of the materials and methods used, the results obtained and the conclusions within each article. The final review included 120 research articles, meta-analyses, systematic reviews and case reports, 1 guideline within a book, and 1 online document (Fig. 1).

## Results

**Fig. 2:**
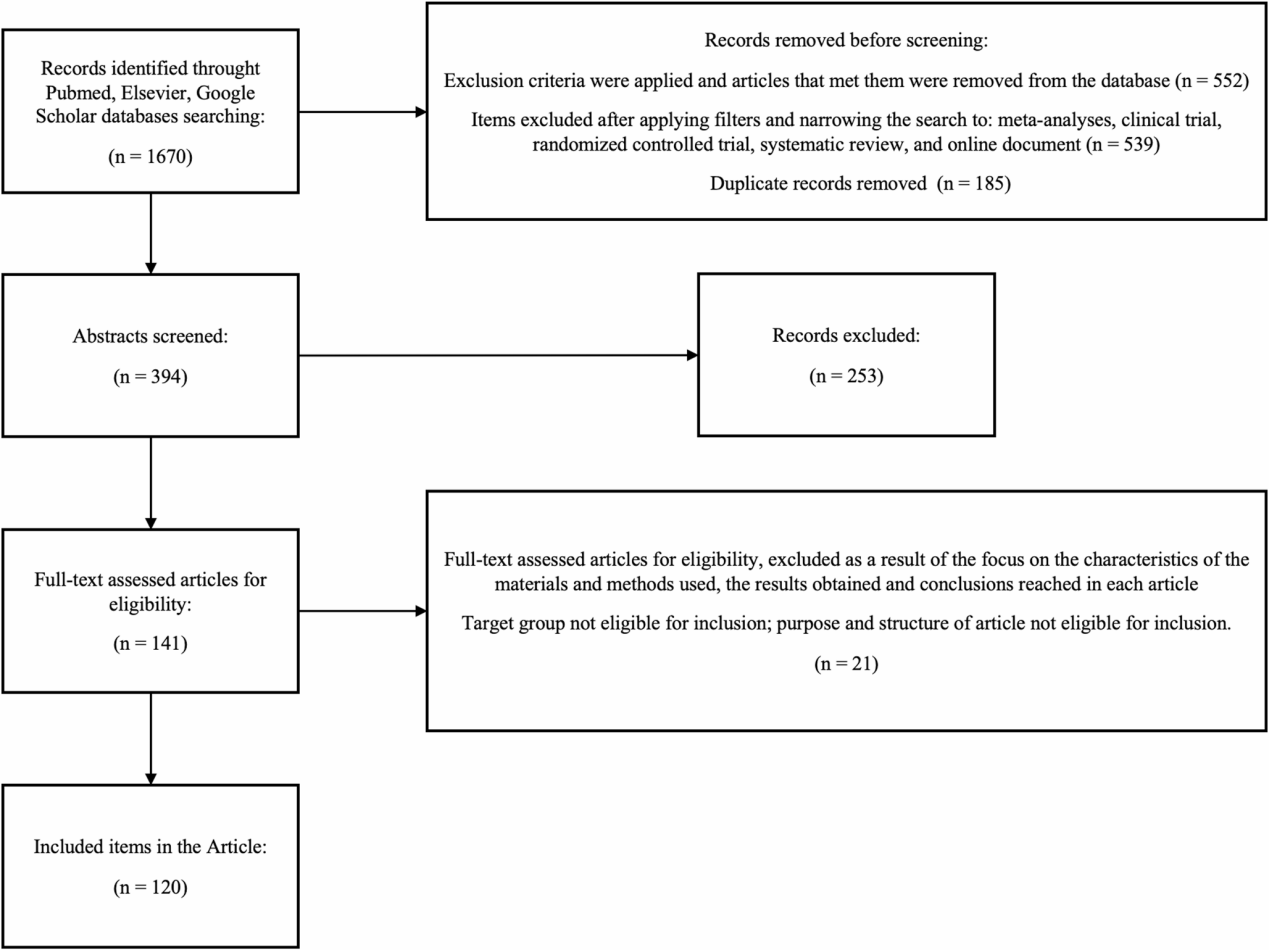
Search strategy and results presented in the Preferred Reporting Items for Systematic Reviews and Meta-analyses (PRISMA)

A primary literature review conducted independently by each author of available articles from PubMed, Google Scholar, and Elsevier databases identified 1670 articles that fit the search pattern. Following the presented search strategy and data collection process, 135 articles were screened and assessed for eligibility. A total of 114 items were included and used in this systematic review. Figure 2 illustrates the search strategies and results, which are presented in their entirety in the Preferred Reporting Items for Systematic Reviews and Meta-analyses (PRISMA) flowchart. It presents the procedure for searching online databases and selecting articles. Table 1 shows the distribution of results within the articles assessed for eligibility that were included in the article, according to the year of publication.

**Table 1:** Distribution of the literature used

Database	Pubmed, Elsevier, Google Scholar, Other		
Included	*N* = 141; *N* = 21 Record excluded during preparation of article		
Total included	*N* = 120		
Types of sources	Books *N* = 1	Articles *N* = 118	Online documents *N* = 1
Year of publication	2007	2019–2024 *N* = 87;	2020
		2014–2018 *N* = 16; Older *N* = 15	

### Definition, pathophysiology and clinical developmental context of the syndrome

According to scientific reports, the occurrence of Guillain-Barré syndrome is usually preceded by an infection, with the acute onset of the disease occurring in most cases following an infectious disease (Shahrizaila et al. 2021). The microorganisms associated with the infection include mainly: *Campylobacter jejuni* (most frequently described due to the best-known mechanism of action) (Shahrizaila et al. 2021), Zika virus, *Mycoplasma pneumoniae*, Epstein-Barr virus and cytomegalovirus (CMV) (Dardiotis et al. 2020). The pathophysiology of the disease is based on damage to peripheral nerves by antibodies against proteins in the myelin sheaths. As a result, of sufficiently large damage to these nerves, cells such as infiltrating macrophages, mast cells, dendritic cells, T cells and neutrophils will contribute to both degenerative and regenerative processes (Hagen and Ousman 2021). Currently, the first meta-analyses showing the association between *Helicobacter pylori* infection and Guillain-Barré syndrome can be observed based on the studies conducted (Dardiotis et al. 2020). Over the last decade, several studies have also been conducted showing the association of HLA-DQB1 genotype with the more frequent occurrence of GBS in patients (Jin et al. 2015). In addition, studies conducted during and after the pandemic are increasingly examining the association of GBS with coronavirus 2 (SARS-CoV-2) infection along with vaccination, especially adenovirus-containing vaccines by comparison with mRNA vaccines (Leonhard et al. 2019).

In reference to the scientific sources used, GBS can be divided based on the clinical picture and the results of nerve conduction studies. There are several main subtypes (Finsterer 2022; Dardiotis et al. 2020; Jin et al. 2015):

- acute axonal motor neuropathy (AMAN).
- acute inflammatory demyelinating polyneuropathy (AIDP).
- acute axonal motor and sensory neuropathy (AMSAN).
- Miller-Fischer syndrome (MFS).

In AMAN, only axons are damaged by the immune system without T cell infiltration (Finsterer 2022). This affects the conduction of nerve signals, causing symptoms similar to demyelinating neuropathy, with differences in more intense loss of sensation, tingling and weakness (Shahrizaila et al. 2021). The pathogenesis of AIDP involves damage to the myelin sheath, which leads to slowing or complete cessation of signal conduction. As a result, there is loss of sensation, tingling, pain or muscle weakness (Shahrizaila et al. 2021; Jin et al. 2015). It is the most frequently observed variant of GBS (Shahrizaila et al. 2021; Jin et al. 2015). Patients with AMAN rapidly demonstrate progressive symmetrical weakness with loss of sensation (Leonhard et al. 2019; Zeng et al. 2019). In MFS (previously classified as an axonal subtype, although most researchers prefer to consider MFS as an independent variant of GBS (Wakerley et al. 2014), ataxia, lack of tendon reflexes, and ophthalmoparesis are observed (Finsterer 2022). Current studies indicate that the cause of GBS is not entirely clear, although there are more and more reports linking *C*. *jejuni* with GBS. They explain that the development of the syndrome through *C*. *jejuni* is explained by “molecular mimicry” between surface lipooligosaccharide (LOS) antigens and ganglioside antigens located on the surface of myelin sheaths or axolemma (Finsterer 2022). A similar mechanism of action to *C*. *jejuni* has been demonstrated in the case of *Mycoplasma pneumoniae*, but it is not clear whether other pathogens cause GBS in a similar way (Finsterer 2022). Currently, there are no specific markers for the assessment of GBS. Among the potential markers considered is the neutrophil to lymphocyte ratio (NLR), which is stress and systemic inflammation indicator as a potential marker reflecting immune dysfunction (Sarejloo et al. 2022). However, the above method has low specificity. Due to the lack of markers, GBS is currently diagnosed based on the clinical picture supported by an interview and physical examination, as well as specialist tests such as electrophysiological, neurological, and cerebrospinal fluid (CSF) examination (Sahin et al. 2017). CSF examinations may show albumin-cytological dissociation, which is considered a characteristic feature of GBS, but it should be noted that CSF examinations may be normal in the initial stages (Sun et al. 2019). It should also be noted that in this syndrome, high levels of glucose in CSF may often occur, which are significantly associated with serious disorders caused by GBS (Gong et al. 2022). This factor is particularly important in patients with abnormal blood glucose levels or directly with diabetes, in whom the disruption of the blood-brain barrier is increased (Gong et al. 2022; Geng et al. 2018). Thus, to summarize, the assessment of GBS is based on the evaluation of seven elements: decreased tendon reflexes, no alternative causes of weakness, monophasic course, bilateral flaccid weakness, CSF (cerebrospinal fluid) cell count < 50 cells/microl, elevated CSF protein concentration and NCS consistent with the subtype (Shahrizaila et al. 2021; Finsterer 2022). Currently, the most effective treatment methods are intravenous immunoglobulin (IVIg) and plasmapheresis. IVIg is intended to inhibit Fc-mediated macrophage activation, prevent complement activation, and prevent antibodies from binding to neuronal bodies, thereby inhibiting further neuronal damage (Verboon et al. 2017). Plasma exchange, on the other hand, is intended to remove neurotoxic antibodies and other inflammatory mediators (Berg et al. 2014). It should be emphasized that treatment strategies should be selected individually based on the standards and recommendations for specific treatment units. Additionally, the study by Doets et al. (Doets et al. 2018) reported evidence for combining complement inhibition with IVIg in Guillain-Barré syndrome to improve outcomes (Doets et al. 2018). According to a 2020 review, monoclonal antibodies, including rituximab, alemtuzumab and eculizumab, have shown preliminary potential, but more clinical trials are still needed to confirm their efficacy (Shang et al. 2021). The prognosis of patients with GBS is difficult to predict due to the high variability of results in individual patients. However, it is assumed that older patients have a worse prognosis compared to patients of younger age. The severity of the syndrome is probably determined in the early phase of the disease (Koningsveld et al. 2002). A simple clinical scoring system, the so-called EGOS, has been developed for use at the bedside of patients with GBS in the acute stage of the disease. It may allow for an accurate prediction of the patient’s chance of independent walking after 6 months. This system can be calculated within the first 2 weeks of the onset of the disease based on the patient’s age, GBS disability score and the presence of previous diarrhea. Based on the developed clinical score, the predicted chance of recovery for individual patients ranges from 1 to 83%. In addition to using EGOS to inform patients about their prognosis, it may have applications in new clinical trials that are more focused on patients with a poorer prognosis (Doorn et al. 2008).

### Epidemiology

The exact annual global incidence of GBS is unknown due to insufficient epidemiological data. It was estimated in 2023 based on studies from North America and Europe performed in previous years and was found to be about 0.81 to 1.91 cases per 100,000 persons per year in adults and about 0.6 per 100,000 persons per year in children (Zheng et al. 2023; Sejvar et al. 2011). In older people, over 80 years of age, it may be 2.7 per 100,000 persons per year (Willison et al. 2016; GeurtsvanKessel et al. 2013; Willison 2005). Studies conducted in East Asia indicate a lower incidence of GBS in these populations (about 1.7/100,000/year in China) compared to Europe or North America (Malek and Salameh 2019; Liou et al. 2016). According to the results of studies obtained so far, the risk of GBS is higher in men than in women, and its incidence increases after the age of 50. It increases by 20% each subsequent decade of life (Sejvar et al. 2011; Malek and Salameh 2019; Liou et al. 2016; Toscano et al. 2020). In some regions of the world, a higher incidence of GBS has been observed in winter than in summer (Malek and Salameh 2019; Webb et al. 2015). Differences in the incidence of GBS worldwide are probably mainly due to local factors present in given populations, such as exposure to pathogenic bacteria and viruses or genetic differences (Toscano et al. 2020; Esposito and Longo 2017; Huang et al. 2015; JACKSON et al. 2014). One of the pathogens associated with GBS is *Campylobacter jejuni*. Its presence is most frequently detected in Asian countries (approximately 25–50% in adults) (GeurtsvanKessel et al. 2013; Islam et al. 2010; Rees et al. 1995). The occurrence of GBS may also be influenced by viruses such as cytomegalovirus or Epstein-Barr virus (GeurtsvanKessel et al. 2013; Jacobs et al. 1998; Mori 2000). The impact of coronavirus 2 (SARS-CoV-2) on the incidence of GBS is still being investigated. In studies conducted in Italy, it was observed that from March to April 2020, the incidence of GBS increased compared to 2019. The rate rose from approximately 0.93/100,000/year to about 2.43/100,000/year (Toscano et al. 2020; Filosto et al. 2021). This increase occurred during the SARS-CoV-2 pandemic, which may suggest a pathogenic relationship between the virus and the increase in the incidence of GBS. According to a study conducted in another region of Italy, a 5.41-fold increase in the incidence of GBS was noted during the pandemic in 2020, compared to previous years (2017–2019) (Toscano et al. 2020; Gigli et al. 2021). This study also focused on March and April (Toscano et al. 2020). The incidence was 0.65/100,000 at that time, compared to approximately 0.12/100,000 in previous years (Toscano et al. 2020; Gigli et al. 2021). In a study conducted in the United States on the association of COVID-19 with GBS, in 2020, 1155 (8.43%) cases of GBS associated with COVID-19 were reported out of 13,705 total cases of GBS (Sharma et al. 2024). It was noted that patients admitted to hospital with GBS associated with COVID-19 required more frequent use of mechanical ventilation. They also had higher mortality during hospital stay than patients admitted with GBS without COVID-19 (Sharma et al. 2024). In a 2021 study, Lina Palaiodimou et al. performed a meta-analysis and systematic review of various studies, which allowed to identify eighteen most relevant (Palaiodimou et al. 2021). They included both hospitalized and non-hospitalized COVID-19 patients with or without GBS. A total of 136,746 cases were recorded, and the incidence of GBS associated with COVID-19 was 15 cases per 100,000 infections (0.15‰) (Palaiodimou et al. 2021). These patients were also observed to have a higher risk of developing demyelinating subtypes of GBS. These results may suggest a preliminary conclusion about the increased probability of a pathogenic association of COVID-19 with the occurrence of GBS morbidity (Palaiodimou et al. 2021).

### GBS vs. COVID-19

**Fig. 3:**
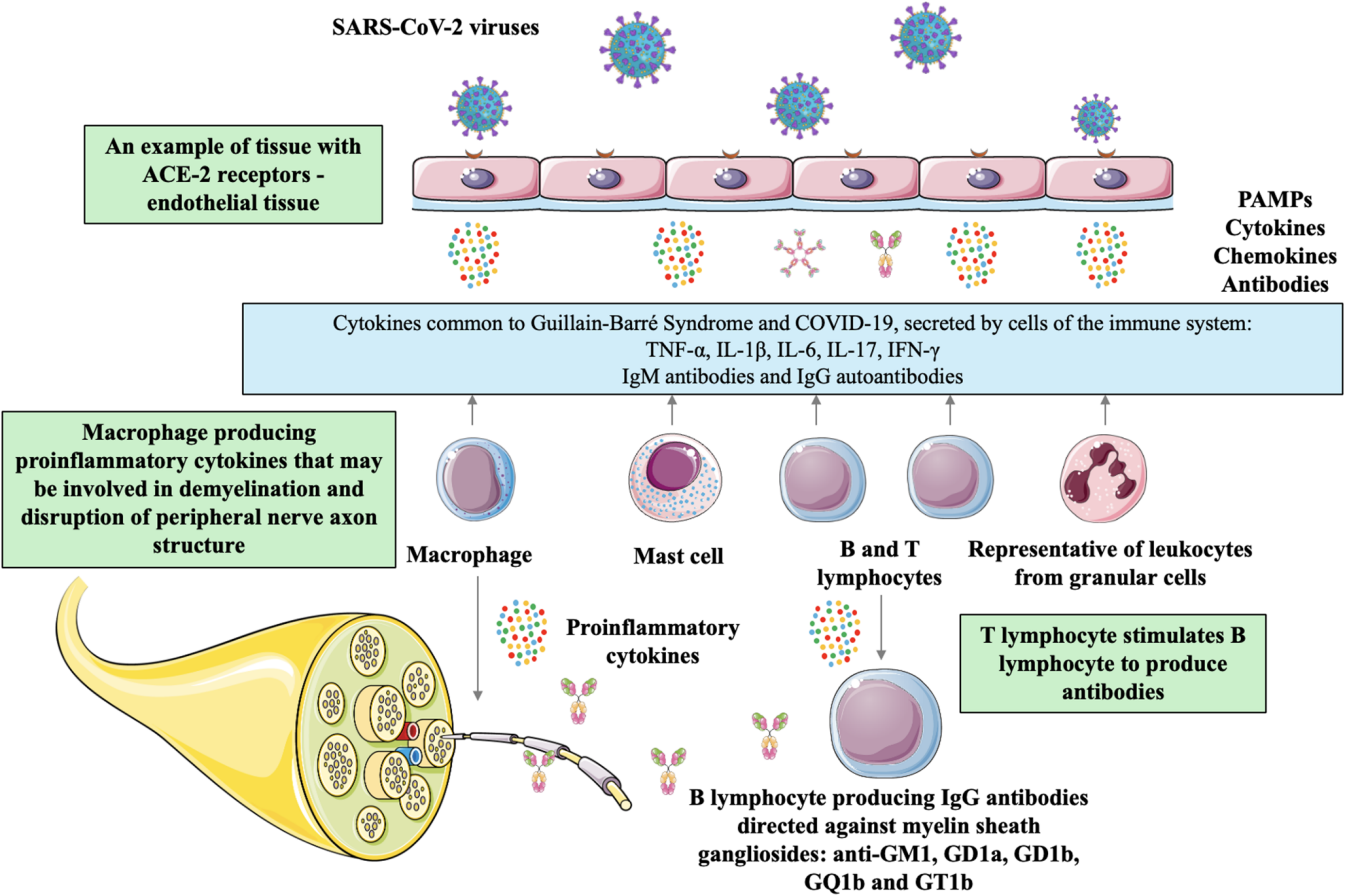
In reference to the literature used, the figure shows the process of SARS-CoV-2 virus binding to the angiotensin-converting enzyme 2 (ACE2) receptor protein using endothelial tissue as an example. In response to pathogen-associated molecular patterns (PAMPs) of the virus, the immune system generates a response of immune system cells including: macrophages, mast cells, B and T lymphocytes and other leukocytes. Large amounts of cytokines, chemokines and IgM antibodies are generated, and then IgG. Regarding the bibliography, only cytokines common to Guillain-Barré syndrome and SARS-CoV-2 virus infection are listed in the figure: TNF-α, IL-1β, IL-6, IL-17 and IFN-γ. Subsequently, T lymphocytes stimulate B lymphocytes to produce IgM antibodies, and later IgG antibodies. Based on molecular mimicry, IgG can become autoantibodies directed against the gangliosides of the myelin sheath: anti-GM1, GD1a, GD1b, GQ1b and GT1b. SARS‑CoV‑2 - severe acute respiratory syndrome coronavirus 2; PAMPs - pathogen-associated molecular patterns; IFN‑γ - interferon‑γ; TNF‑α - tumor necrosis factor‑α; IL - interleukin. Accordining to (Bentley et al. 2022; Zheng et al. 2023; Palaiodimou et al. 2021; Ramasamy and Subbian 2021; Lee and Shin 2020; Lee et al. 2020; Chitsamankhun et al. 2024; Hussain et al. 2020; Fantini et al. 2020). Figure 3 was created using the Servier Medical Art Commons Attribution 4.0 Unported Licence http://smart.servier.com. https://creativecommons.org/licenses/by/4.0/

The SARS-CoV-2 virus is particularly transmitted in crowded, isolated spaces with limited air circulation or as a result of prolonged contact with a person with COVID-19 disease. An infected person can transmit the virus through droplets when speaking, breathing, coughing or sneezing. Other routes of spreading the virus include aerosol and contact of the eyes, nose or mouth with a surface contaminated with the virus, which is defined as fomite transmission (Rathee et al. 2020; Sharma et al. 2021; Cai et al. 2020). In order to become infected with SARS-CoV-2, in most cases the presence of virus particles in the order of hundreds or more is necessary (Karimzadeh et al. 2021; Basu 2021). Another possible method of virus transmission is the fecal-oral route. However, despite the proven occurrence of RNA aerosols in the immediate vicinity of toilets and the presence of SARS-CoV-2 RNA in a rectal swab, this route remains unexplained (Harrison et al. 2020; Xiao et al. 2020; Liu et al. 2020; Xu et al. 2020). Additionally, it has been suggested that the SARS-CoV-2 virus may infect the central nervous system *via* the olfactory pathway of the olfactory nerve, general circulation or pulmonary peripheral neurons (Bentley et al. 2022; Das et al. 2020). The olfactory bulb allows various viruses to reach the brain, from where they can reach specific regions, such as the brainstem or thalamus (Bentley et al. 2022; Zhou et al. 2020a, b; Gutiérrez-Ortiz et al. 2020). The combination of the virus fusion protein (S-spike) with the angiotensin-converting enzyme 2 (ACE2) receptor protein enables the infiltration of SARS-CoV-2 particles into target cells (Rombel-Bryzek et al. 2023). Secondary tissue injury of organs with low ACE2 production in COVID-19 patients may indicate a possible role of other receptors in virus fusion. Studies have shown the potential role of other proteins in virus fusion: ADAM17, CD147 and GRP78 (Simon et al. 2021; Alipoor and Mirsaeidi 2022; Verdecchia et al. 2020; Kirtipal et al. 2022). SARS-CoV-2 fusion can occur *via* two routes, endocytic, which is the basic pathway, and surface, using transmembrane serine protease 2 (TMPRSS2) (Heurich et al. 2014; Li et al. 2024). Thanks to the receptor binding domain (RBD) of the S protein, Spike has the ability to bind to ACE2, which allows cathepsins and TMPRSS2 to separate at the S1/S2 site. Separation of S1 allows for the exposure of the S2 subunit domain and the initiation of fusion (Harrison et al. 2020; Li et al. 2024; Nithya Shree et al. 2024; Zhou et al. 2020a, b; Wu et al. 2020; Hoffmann et al. 2020; Wrapp et al. 1979). After attachment, the SARS-CoV-2 virus undergoes endocytosis. The virus’s connection with the lysosome membrane is facilitated by cathepsins, which allow the introduction of viral RNA into the cytoplasm. An alternative route of virus entry assumes a direct connection of the cell membrane with the SARS-CoV-2 envelope, mediated by TMPRSS2 (Nazerian et al. 2021; Jackson et al. 2022; Eijk et al. 2021). High production of TMPRSS2 and ACE2 in the gastrointestinal tract, primarily on the surface of intestinal enterocytes, may indicate an anorectal method of SARS-CoV-2 transmission (Hoffmann et al. 2020; Hamming et al. 2004; Ziegler et al. 2020; Sun et al. 2021; Zhang et al. 2020). The key sites of SARS-CoV-2 expansion after entering the respiratory system are macrophages, pneumocytes and endothelial cells (Harrison et al. 2020; Hamming et al. 2004). Pathogen-associated molecular patterns (PAMPs) of the virus after cell infiltration bind to pattern recognition receptors (PRRs). According to selected literature, these include Toll-like receptors (TLRs), such as TLR8, TLR7 and TLR3 (Ramasamy and Subbian 2021; Kawasaki and Kawai 2014). Additionally, PAMP can also be bound by protein kinase C (PKC) and melanoma differentiation-associated protein 5 (MDA5) (Ramasamy and Subbian 2021; Kawasaki and Kawai 2014). PRRs initiate the immune response. Due to the induction of the cell signaling pathway, neutrophil mobilization, macrophage activation and induction of interferon regulatory factor (IRF) gene transcription occur (Li et al. 2024). As a result, the production of type 1 interferons (IFN-I) is initiated (Ramasamy and Subbian 2021; Thiel and Weber 2008; Fitzgerald et al. 2003). It is also possible to stimulate the nuclear transcription factor NF kappa B (NF-κB) pathway due to the detection of PAMPs in the cell (Hadjadj et al. 2020; Kim et al. 2021). As a consequence, the immune response will be enhanced due to the production of proinflammatory cytokines, such as interleukin-6 (IL-6) and tumor necrosis factor alpha (TNF-α) (Hadjadj et al. 2020; Kim et al. 2021). The components of SARS-CoV are able to evade the non-specific immunity of the body by counteracting the IFN response (Grifoni et al. 2020; Park et al. 2020; Chan et al. 2020; Clementz et al. 2010). Based on the selected literature, these include the membrane protein (M), spike protein (S), nucleocapsid protein (N), non-structural proteins NSP14, NSP13 and NSP1, and open reading frame 3b (ORF3b), ORF6, ORF7 and ORF8 (Ramasamy and Subbian 2021; Grifoni et al. 2020; Park et al. 2020; Chan et al. 2020; Clementz et al. 2010; Li et al. 2020; Konno et al. 2020). Therefore, the immune response to SARS-CoV-2 is associated with a weakened IFN response (Ramasamy and Subbian 2021; Hu et al. 2021; Hussman 2020). Impaired activation of IFN-I has been shown to increase the mobilization of mast cells, macrophages and monocytes, lymphocytes and numerous other leukocytes in laboratory models of COVID-19 mice with human ACE2 (hACE2). That results in an excessive inflammatory response (Fig. 3) (Ramasamy and Subbian 2021; Lee and Shin 2020; Lee et al. 2020; Chitsamankhun et al. 2024). In addition, the reduction of ACE2 level, through decreased production and binding to the virus, causes an increase in the amount of angiotensin II (AngII) (Jiang et al. 2022). AngII has the ability to stimulate the production of, among others, TNFα, soluble IL-6 receptor (sIL-6Rα) and epidermal growth factor receptor (EGFR) ligands. Which are able to stimulate NF-kB, leading to its overactivity (Jiang et al. 2022). As a result, there is an increased production of proinflammatory cytokines, which in consequence may cause a cytokine storm (Jiang et al. 2022). Cytokine storm is a secondary increased inflammatory response caused by immune dysfunction associated with excessive synthesis of proinflammatory cytokines. It is an important element related to the symptoms and severity of COVID-19 (Zanza et al. 2022). Ying Chi et al. (Chi et al. 2020) examined the serum of COVID-19 patients to assess the amount of chemokines and cytokines (Chi et al. 2020). Their analysis showed increased amounts of chemokines and 27 cytokines compared to uninfected individuals. These included IL-18, IL-17, IL-15, IL-13, IL-10, IL-9, IL-8, IL-7, IL-6, IL-2Rα, IL-2, IL-1ra, IL-1β, basic FGF, HGF, PDGF-BB, VEGF, GRO-α, IP-10, MCP-1 and MIG, G-CSF, M-CSF, IFN-α2, IFN-γ, TNF-α and TRAIL (Chi et al. 2020). Numerous cytokines (TNF-α, IL-1β, IL-6, IL-17, IFN-γ) are involved in the pathogenesis of GBS and are also involved in the development of COVID-19 disease (Fig. 3) (Bentley et al. 2022; Palaiodimou et al. 2021; Hussain et al. 2020).

It is suspected that the SARS-CoV-2 virus, due to increased cytokine production and autoimmune dysfunction, is able to initiate the occurrence of GBS (Bentley et al. 2022; Yu et al. 2006). It is assumed that the consequence of the cytokine storm associated with COVID-19 may be demyelination and disruption of the blood-brain barrier function (Palaiodimou et al. 2021; Hussain et al. 2020; Tsivgoulis et al. 2020). The ACE2R-related SARS-CoV-2 S1 protein is absorbed by the activated APC protein C, and then presented to T lymphocytes, which stimulate B lymphocytes to produce antibodies against gangliosides (Censi et al. 2024). In 17.6% of patients, the presence of antiganglioside antibodies was found (Censi et al. 2024; Butler et al. 2022). According to the selected literature, these included anti-GM1, GD1a, GD1b, GQ1b and GT1b antibodies (Fig. 3) (Zheng et al. 2023; Civardi et al. 2020). T lymphocytes release IFN and damage the blood-brain barrier by producing IL-1, IL-6 and TNF-α, thus fueling the immune response. Macrophages are mobilized, which produce pro-inflammatory cytokines, leading to demyelination and disruption of the axonal structure of peripheral nerves (Zheng et al. 2023). Additionally, the presence of antiganglioside antibodies in COVID-19 patients suggests the possibility of “molecular mimicry”. The presence of SARS-CoV-2 may result in damage to peripheral nerves, due to cross-reaction between viral proteins and cell gangliosides. As a result, an immune response occurs, which may ultimately lead to the development of GBS (Zheng et al. 2023; Fantini et al. 2020). Therefore, control of cytokine function is suggested as a therapy against COVID-19 and GBS (Palaiodimou et al. 2021). In a meta-analysis conducted by Stefano Censi et al., the median time interval between SARS-CoV-2 infection and the development of GBS was 14 days (Censi et al. 2024). Vitória Pimentel et al. described the predominant symptoms in patients with COVID-19 as dyspnea, fever, and cough (Pimentel et al. 2023). The key symptoms of GBS include: decreased or absent reflexes, paresis or paralysis of facial muscles, general weakness, hypoesthesia, and paresthesia (Pimentel et al. 2023). The clinical picture of GBS in patients with COVID-19 does not differ significantly from the symptoms that occur in cases caused by other factors (Pimentel et al. 2023; Jasti et al. 2016). A systematic review by Vitória Pimentel et al. also revealed that hydroxychloroquine and/or antibiotics were most commonly used in the treatment of COVID-19. In turn, for Guillain-Barré syndrome, the most common treatment method was the use of intravenous immunoglobulins (IVIG) (Pimentel et al. 2023). An alternative treatment method is plasma exchange, which eliminates antibodies toxic to the nervous system and other inflammatory factors (Bentley et al. 2022; Berg et al. 2014). Studies have shown that this therapy achieves the best results when it is carried out within 2–4 weeks of the first symptoms of weakness (Bentley et al. 2022; Berg et al. 2014).

## Discussion

### COVID-19 vaccinations and GBS

To date, studies are being conducted on the link between COVID-19 vaccinations and the occurrence of GBS. Many clinically important studies have been conducted, which emphasize that the action of vaccines may affect the development of GBS, contributing to the occurrence of symptoms characteristic of this disease. Single cases of Guillain–Barré syndrome have been reported after vaccination against hepatitis B, tetanus, polio, meningitis, rabies, and oral adenovirus (Introna et al. 2021). A potential association between GBS and vaccination was discussed after the 1976 H1N1 influenza vaccination campaign when an increased number of GBS cases was reported (Introna et al. 2021). Similar concerns were noted during the 2009 influenza immunization program, although the supporting evidence was less convincing (Introna et al. 2021). Several investigators have suggested that these reports may reflect coincidental temporal associations rather than a direct causal relation (Introna et al. 2021). Cautious interpretation of such data is essential to prevent unfounded concerns about vaccine safety. Vaccines used against COVID-19 are mRNA vaccines (Comirnaty (BNT162b2) by Pfizer&BioNTech, Spikevax (mRNA 1273) by Moderna), vector vaccines (ChAdOx1 nCoV-19 - AstraZeneca, Ad26.COV.2-S - Janssen) and recombinant vaccines - NVX-CoV2373 by Novavax (two-dose) (Zheng et al. 2023). In Europe, the following are still under review: the vector vaccine - Sputnik V (Gam-COVID-Vac) and the inactivated SARS-CoV − 2 vaccine (Vero cells). mRNA vaccines contain genetic information that, after entering the cell cytoplasm, is designed to trigger the mechanisms responsible for identifying the spike protein present on the surface of the SARS-CoV-2 virus (Zheng et al. 2023; Scendoni et al. 2021). After recognizing the foreign antigen (spike glycoprotein), the body produces appropriate antibodies, which allows for faster killing of the COVID-19 virus during its re-entry and prevents the development of the disease (Zheng et al. 2023; Scendoni et al. 2021). The immune system response may trigger autoimmune processes in some patients leading to the development of GBS, due to the production of autoantibodies directed against myelin (Chowdhury and Chowdhury 2023). mRNA vaccines against SARS-CoV-2 use viral vectors in the form of adenoviruses, incapable of replication, to induce a targeted immune response. Adenovirus is necessary for its induction, which provides the genetic material of the S protein antigen (Scendoni et al. 2021; Waheed et al. 2021). It is assumed that the development of GBS, in this case, may be due to an immunological cascade leading to the production of specific antibodies and to the occurrence of cross-reactions with proteins present on nerves (Zheng et al. 2023). The NVX-CoV2373 vaccine by Novavax is a recombinant, subunit vaccine. This means that it has a ready antigen - the entire S protein and the Matrix-M adjuvant, which is intended to enhance the immune response. According to a study published in March 2024, the highest number of GBS cases occurred after the AstraZeneca (Vaxzervia) vaccine (48 people out of 103 cases) (Sah et al. 2024a, b). The Pfizer vaccine came second (23 people), and the Johnson & Johnson and Moderna vaccines came third (8 people). The time to onset of the first symptoms of the disease after the AstraZeneca vaccine was on average 13 ± 11 days IQR (interquartile range). The highest number of adverse events was reported after the first dose of the vaccine, and the lowest after the 3rd dose (2 cases) (Sah et al. 2024a, b). Another study from 2023, conducted by Selia and Samia Chowdhury, analyzed cases in which GBS occurred after the COVID-19 vaccine (Chowdhury and Chowdhury 2023). As in the previous study, the highest number of cases occurred after the use of the AstraZeneca (Vaxzevria) vaccine − 36 out of 67 people. In the case of the Pfizer vaccine, there were 12 people with GBS. After the use of the Johnson & Johnson vaccine − 6 people, and Moderna − 5 people (Chowdhury and Chowdhury 2023). The time between the administration of the vaccine and the appearance of the first symptoms of the disease was about 13 days (12.67). GBS most often developed after vaccination in people aged 45–65 years (mean 51.66 years) and was dominant in men (61.2%, and women − 38.8%) (Chowdhury and Chowdhury 2023). In the treatment of this form of GBS, plasmapheresis, intravenous immunoglobulin (IVIG), and steroids were used (Chowdhury and Chowdhury 2023). In 2021, between January and October, the English national database noted a 2.04-fold increase in the risk of developing GBS within 28 days of administration following the AstraZeneca vaccine. No similar results were observed following the Pfizer vaccine (Keh et al. 2023). Based on data provided to the Mexican Ministry of Health, studies were conducted from December 24, 2020, to March 19, 2021, on the effect of the BNT162b2 vaccine on the development of GBS. It was shown that patients who received this type of vaccine rarely developed GBS (García-Grimshaw et al. 2021). Symptoms of the disease appeared only after the first dose of the vaccine (García-Grimshaw et al. 2021). The Vaccine Adverse Event Reporting System (VAERS) reported 130 cases of GBS in July 2021, most likely following vaccination with Ad26.COV2.S (Janssen) (Woo and Pieters 2021). The median age of those who presented with symptoms was 56 years. GBS occurred more frequently in men (59.7%) than in women after vaccination (Woo and Pieters 2021). A 2023 systematic review compared mRNA vaccines and Ad.26.COV2.S (Janssen) in terms of the incidence of GBS development over a time period ranging from 1 to 21 days (Hanson et al. 2022). The incidence of GBS following mRNA vaccines was observed to be 1.3 per 100,000 person-years, compared with 32.4 per 100,000 person-years for the Ad.26.COV2.S vaccine. This indicates that the Ad.26.COV2.S vaccine has a higher risk of developing GBS than mRNA vaccines (Hanson et al. 2022). It has been noted that, unlike the classic form of GBS, the form of GBS occurring after COVID-19 vaccination is associated with the occurrence of more severe symptoms, and patients more often develop hemiparesis (Zheng et al. 2023). In a study conducted by Lay Khoon Loo et al., in 2021, it was found that in 14 out of 16 people with acute polyradiculoneuropathy, symptoms of the disease occurred after the first dose of the AstraZeneca vaccine (87.5%) (Loo et al. 2022). Initial symptoms appeared within 4 weeks of the administration of the first dose of this vaccine. These were mainly facial weakness (56.3%) and fingertips (50%), bilateral weakness, or distal paresthesia (GBS variant) (Loo et al. 2022). They occurred more often after vaccination than in patients with GBS from 2005 to 2019. The dominant clinical subtype was classic GBS (75%) (Loo et al. 2022). In 2021, Shih-Chieh Shao et al. performed a systematic review, which allowed the discovery of 39 cases of GBS occurring worldwide after vaccination (including 7 cases from Mexico, 10 cases in India, and 1 in Taiwan) (Shao et al. 2021). The mean age of onset was 57.8 years, and the disease developed more often in men (56.4%). Patients in the largest number received the ChAdOx1-S vaccine (25/39), followed by BNT162b2 (12/39) and Ad26.COV2.S (1/39). The incidence of GBS after vaccination ranged from 1.8 to 53.2 cases/1 million doses (Shao et al. 2021). The first symptoms appeared approximately 11.3 days after vaccination. The most common of these are muscle pain (12/39), paresis (5/39), quadriplegia (22/39), paresthesia (28/39), and facial paralysis (23/39) (Shao et al. 2021). GBS is considered a rare adverse effect of COVID-19 vaccines. In the context of a mass vaccination program involving approximately 5 billion people, statistical analyses suggest that purely by chance between 10,000 and 20,000 cases of GBS may occur in any 10-week period (Introna et al. 2021). This timeframe includes the interval between the two doses of the vaccine. As a result, thousands of cases of GBS that are not related to the vaccine will likely coincide with COVID-19 immunization (Introna et al. 2021). It is therefore crucial for public health authorities and the scientific community to take proactive measures to prevent unwarranted vaccine hesitancy. These can result from misunderstandings or misattributions related to these coincident events (Introna et al. 2021). Providing clear communication about the epidemiological evidence and accurate risk assessments is vital to maintaining public confidence in vaccination programs. Some authors suggest that viral vector vaccines may trigger systemic immune activation through mechanisms of molecular mimicry (Introna et al. 2021). These processes could help explain the previously proposed link between COVID-19 vaccination and Guillain-Barré syndrome. This occurs by inducing an immune response with specific characteristics, which might lead to the unique immune signature observed in these cases (Introna et al. 2021). Studies and analyses conducted so far indicate a possible relationship between the action of SARS-CoV-2 vaccines and the development of GBS. However, there is still a need to perform further clinical studies.

## Conclusions

In relation to the discussion conducted within the applied literature within this systematic review, a statistical association between SARS-CoV-2 infection and the occurrence of Guillain-Barré syndrome can be confirmed. The presented scientific evidence also confirmed with high probability the influence of immunological processes accompanying the infection on the pathogenesis of this syndrome. However, the current state of knowledge on this topic requires further development in order to more precisely determine the pathophysiological process of GBS both in the context of COVID-19 and other infectious diseases. In terms of the applied literature, Guillain-Barré syndrome is also included among the rare adverse effects of vaccines adenovector and mRNA vaccines against the SARS-CoV-2 virus infection in the development of its different types. The studies and analyses conducted so far indicate the potential possibility of a relationship between the action of COVID-19 vaccines and the development of GBS due to immunization and the production of antibodies against myelin sheaths.

## Data Availability

No datasets were generated or analysed during the current study.

## Abbreviations

SARS‑CoV‑2: Severe acute respiratory syndrome coronavirus
GBS: Guillain-Barré syndrome
